# Role of insulin receptor substrates in the progression of hepatocellular carcinoma

**DOI:** 10.1038/s41598-017-03299-3

**Published:** 2017-07-14

**Authors:** Yoshitaka Sakurai, Naoto Kubota, Iseki Takamoto, Atsushi Obata, Masahiko Iwamoto, Takanori Hayashi, Masakazu Aihara, Tetsuya Kubota, Hiroshi Nishihara, Takashi Kadowaki

**Affiliations:** 1 Department of Diabetes and Metabolic Diseases, Graduate School of Medicine, The University of Tokyo, Tokyo, Japan; 2 Department of Clinical Nutrition Therapy, The University of Tokyo, Tokyo, Japan; 3 Clinical Nutrition Program, National Institute of Health and Nutrition, National Institutes of Biomedical Innovation, Health and Nutrition, Osaka, Japan; 4 Laboratory for Metabolic Homeostasis, RIKEN Center for Integrative Medical Sciences, Kanagawa, Japan; 5 Department of Diabetes, Endocrinology and Metabolism, Kawasaki Medical School, Kurashiki, Okayama, Japan; 6 Department of Translational Pathology, Hokkaido University Graduate School of Medicine, Sapporo, Hokkaido, Japan; 7 Translational Research Laboratory, Hokkaido University Hospital, Clinical Research and Medical Innovation Center, Sapporo, Hokkaido, Japan

## Abstract

Several cellular signaling pathways, including insulin/IGF signaling, are known to be activated in hepatocellular carcinoma (HCC). Here, we investigated the roles of insulin receptor substrate (Irs) 1 and Irs2, both of which are the major molecules to be responsible for transducing insulin/IGF signaling in the liver, in the development of HCC by inducing chemical carcinogenesis using diethylnitrosamine (DEN) in mice. The Irs1 mRNA and protein expressions were upregulated in the tumors, along with enhanced insulin signaling. Liver-specific Irs1-knockout (*LIrs1KO*) mice exhibited suppression of DEN-induced HCC development, accompanied by reduced cancer cell proliferative activity and reduced activation of Akt. Gene expression analyses revealed that the tumors in the DEN-treated *LIrs1KO* mice showed modest metabolic alterations during hepatocarcinogenesis as well as decreased inflammation and invasion potentials. On the other hand, liver-specific Irs2-knockout (*LIrs2KO*) mice showed a similar pattern of HCC development to the DEN-treated control wild-type mice. Based on the knowledge that Wnt/β-catenin signaling is activated in HCC, we focused on Wnt/β-catenin signaling and demonstrated that Irs1 expression was induced by Wnt3a stimulation in the primary hepatocytes, associated with insulin-stimulated Akt activation. These data suggest that upregulated Irs1 by Wnt/β-catenin signaling plays a crucial role in the progression of HCC.

## Introduction

Hepatocellular carcinoma (HCC) is one of the major cause of cancer-related death worldwide^1^. There are diverse risk factors for the development of HCC, including viral hepatitis, alcoholic liver disease, carcinogen exposure and metabolic liver diseases. Regardless of the etiology, several important cellular signaling pathways are known to be activated during hepatocarcinogenesis, such as the RAF/MEK/ERK pathway, Wnt/β-catenin pathway, and phosphatidylinositol-3 kinase (PI3K)/Akt/mammalian target of rapamycin (mTOR) pathway^2^. In addition, recent epidemiologic studies have revealed that type 2 diabetes^3^ and obesity^4^ are strongly associated with the risk of development of HCC. Both type 2 diabetes and obesity are characterized by hyperinsulinemia associated with insulin resistance. Although enhanced inflammation in obesity has promoted the development of HCC^5^, the mechanism underlining the association between insulin signaling and hepatocarcinogenesis is still unclear.

After insulin and insulin-like growth factor (IGF)1 bind to their receptors, autophosphorylation of tyrosine residues in the intracellular subunit of each of the receptors causes docking and phosphorylation of the insulin receptor substrates, followed by activation of the downstream kinase cascades^6^. Although there are six different kinds of insulin receptor substrates with different tissue-specific distributions, insulin receptor substrate (Irs1)1 and Irs2 are the major isoforms that are thought to be responsible for transducing insulin signaling in the liver^7^. Irs1 expression has been shown to be upregulated in human HCC^8^, and significant relationship has been reported between the Irs1 expression level and the tumor size^9^. Enhanced Irs1expression has also been observed in HCCs induced by intrahepatic pancreatic islet transplantation in diabetic rats^10, 11^. In addition, higher expression levels of phosphorylated Irs1 have been detected in human HCCs, which have also been shown to be negatively correlated with the patient survival rates^12^. Moreover, not only Irs1, but also Irs2 has been shown to be upregulated in both human and murine HCC^8, 13^. However, the causal relationship between hepatocarcinogenesis and hepatic Irs1 or Irs2 is still not well understood.

In the present study, we investigated the characteristics of HCC development in liver-specific Irs1-knockout (*LIrs1KO*) and liver-specific Irs2-knockout (*LIrs2KO*) mice treated with diethylnitrosamine (DEN), to understand the specific roles of Irs1 and Irs2 in hepatocarcinogenesis. Because DEN-induced HCC in mice has the similar histology and genetic signature to human HCC^14^, this mouse model is widely used as a HCC model^15^. In a recent study, we demonstrated that *LIrs1KO* mice were protected from high-fat-diet-induced hepatic steatosis^16^. On the other hand, these KO mice fed standard chow exhibited relatively normal insulin levels and hepatic TG contents^7^. A high-fat diet was not adopted based on these studies, in order to ensure elimination of these otherwise confounding factors in HCC development. Herein, we report that insulin signaling was activated in HCC induced by DEN, accompanied by increased Irs1 expression. Deletion of Irs1, but not Irs2, in the hepatocytes significantly suppressed the HCC development. These data suggest that insulin signaling, especially Irs1-mediated signaling, plays an important role in hepatocarcinogenesis.

## Results

### Irs1 is upregulated in DEN-induced HCC

**Figure 1:**
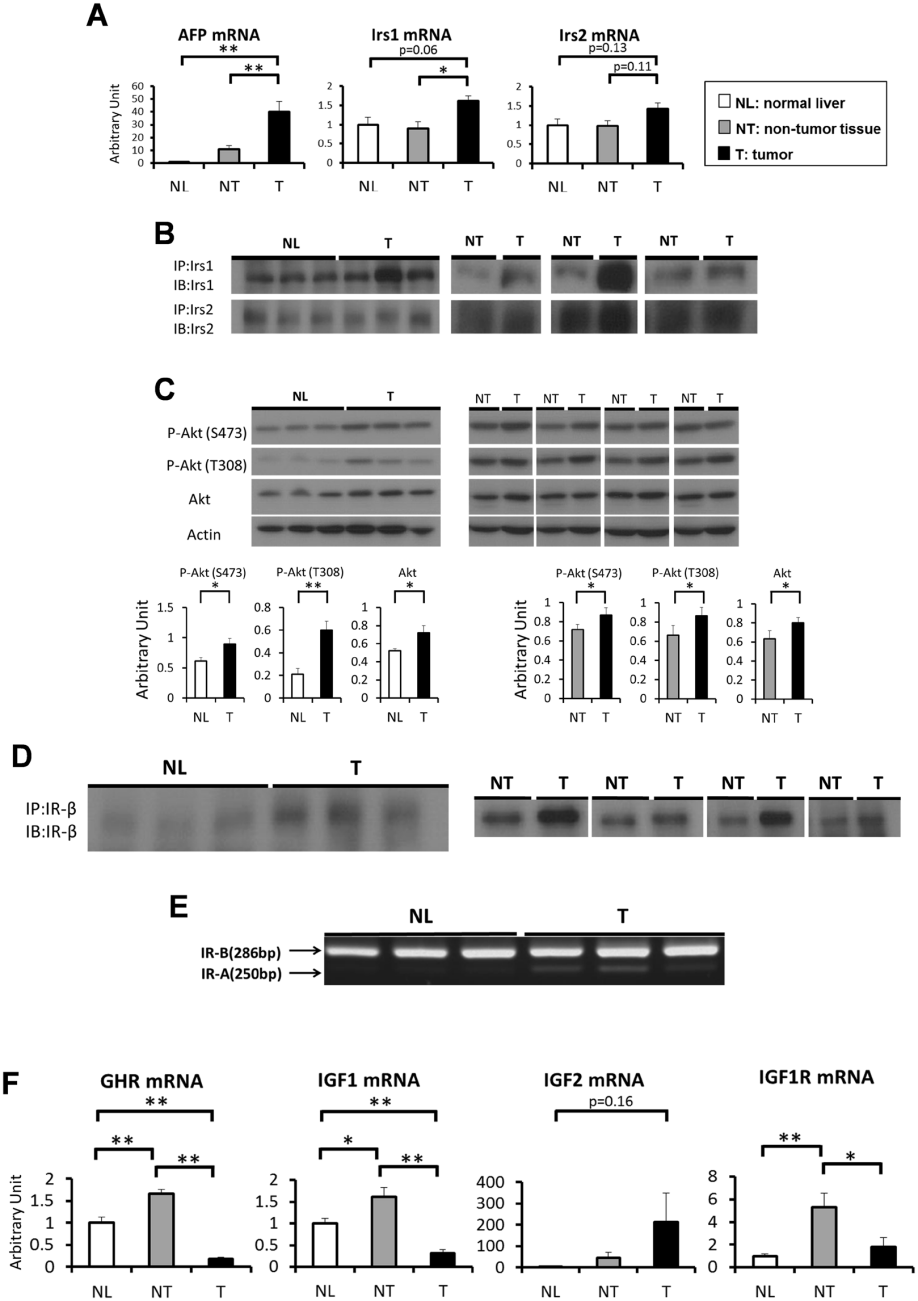
Irs1 is upregulated in DEN-induced HCC. (**A**) Expression levels of AFP, Irs1 and Irs2 genes in each group of mice (n = 7). (**B**) Western blot analysis of Irs1 and Irs2 protein expression levels in NL and T, or NT and T. Lysates from each group were immunoprecipitated (IP) and subsequently immunoblotted (IB) with the indicated antibodies. (**C**) Phosphorylation and protein expression levels of Akt. Representative immunoblot analysis for Akt and p-Akt (upper panel). Results were quantified and normalized to Actin (lower panel) in NL and T (n = 8), or NT and T (n = 8, paired t test). (**D**) Protein expression levels of Insulin receptor (IR). Lysates from each group were immunoprecipitated (IP) and subsequently immunoblotted (IB) with the antibody against IR-β. (**E**) The Insulin receptor (IR) isoforms **A** and **B** are indicated by an arrow. Total RNA was extracted from each group and cDNA was synthesized. After PCR experiments using primers for the flanking exons 10 and 12, reaction products were resolved on 2% agarose gels. (**F**) Expression levels of GHR, IGF1, IGF2 and IGF1R genes in each group (n = 7). Values are the means ± SEM of data obtained from each group. NL, normal liver tissues; NT, non-tumor tissues; T, tumors. *P < 0.05. **P < 0.01.

Ten months after administration of DEN or normal saline to wild-type mice (C57BL/6 J), the expression levels of Irs were compared between the HCC tumors and the surrounding non-tumor tissues as also normal liver tissue. The lesions macroscopically recognized as tumors exhibited high AFP mRNA expression levels, suggesting that HCC was induced as expected by single DEN administration and macroscopic separation between the tumors and non-tumor tissues was appropriate (Fig. 1A). While the Irs1 mRNA expression levels were significantly elevated in the tumors, Irs2 mRNA expression levels were not significantly different among these groups (Fig. 1A). Comparative analysis between the tumors and matched non-tumor tissues in each individual mouse revealed that Irs1 mRNA expression was indeed significantly upregulated in the tumors (Supplementary Figure S1). While a tendency towards increased tumor Irs2 mRNA expression was also noted, the increase in the tumor: non-tumor tissue expression ratio was significantly higher for Irs1 mRNA than for Irs2 mRNA (Supplementary Figure S1). Increased Irs1 protein expression was also observed in the tumors (Fig. 1B), with elevation of the Akt protein and phosphorylation levels (Fig. 1C). In addition, insulin receptor (IR) protein expression was upregulated (Fig. 1D), and expression of the IR-A alternative splicing isoform related to high sensitivity to insulin^17^ and IGF2^18^ was also observed in the HCCs (Fig. 1E). Because Irs1 and Irs2 are also associated with the transduction of signals from IGF1 and IGF2, besides those from insulin, the expression levels of molecules related to IGF signaling were analyzed. While the mRNA expression levels of GHR, IGF1 and IGF1R were increased in non-tumor tissues, they were downregulated in the tumors (Fig. 1F). The IGF2 mRNA expression levels were not significantly different among these groups. These results suggest that Irs1 expression was upregulated in DEN-induced HCC, associated with increase in insulin signaling.

### Loss of Irs1 in the hepatocytes suppressed DEN-induced hepatocarcinogenesis

**Figure 2:**
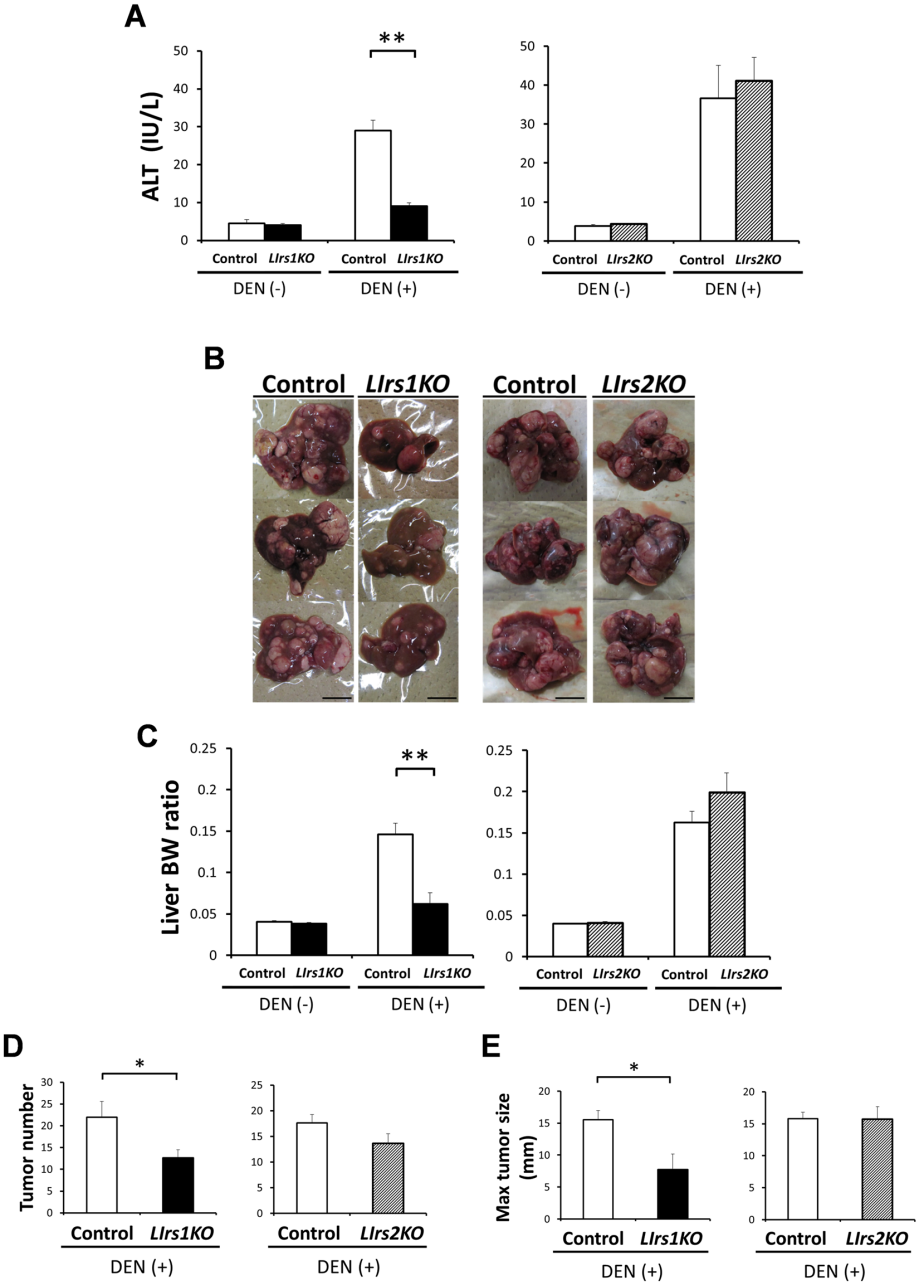
Loss of Irs1 in the hepatocytes suppressed DEN-induced hepatocarcinogenesis. Ten months after DEN administration, *LIrs1KO* and *LIrs2KO* mice were compared with respective control (*Irs1* ^*lox/lox*^ and *Irs2* ^*lox/lox*^) wild-type mice. (**A**) Serum levels of ALT. The mice in each group treated with not DEN but normal saline were used as negative controls. (**B**) Gross appearances of representative livers with tumors in each DEN-treated mouse group. Scale bar, 10 mm. (**C**) The liver: body weight ratio, (**D**) liver tumor numbers (>1 mm) and (**E**) maximal tumor size (diameter in mm) on the surface were determined in each DEN-treated mouse group. Values are the means ± SEM of data obtained from each group. DEN(−), n = 6–9; DEN(+), n = 11–12. *P < 0.05. **P *<* 0.01.

To elucidate the roles of Irs1 and Irs2 in HCC development, *LIrs1KO*, *LIrs2KO* mice and the respective control (*Irs1* ^*lox/lox*^ and *Irs2* ^*lox/lox*^) mice were injected with 25 mg/kg of DEN at the age of 15 days. Ten months after this DEN administration, all of the mice developed numerous visible HCC nodules, while no HCC nodules were detected on either macroscopic or microscopic examination in any of the mouse groups treated with normal saline (Supplementary Figure S2). DEN-treated *LIrs1KO* mice showed lower serum levels of alanine aminotransferase (ALT) than the DEN-treated control wild-type mice, whereas the levels were similar between the DEN-treated *LIrs2KO* mice and DEN-treated control wild-type mice (Fig. 2A). Gross appearances of the livers revealed that the development of DEN-induced HCC was dramatically inhibited by hepatocyte-specific ablation of Irs1, whereas gross appearances of DEN-induced HCC development were comparable in *LIrs2KO* mice and control mice (Fig. 2B). When the tumor burden in the livers from each DEN-treated mouse group was determined by evaluating the liver: body weight ratio, the tumor burden was found to be much lower in the DEN-treated *LIrs1KO* mice as compared to that in the DEN-treated control mice (Fig. 2C). Macroscopic examination revealed decreased multiplicity and sizes of the HCC tumors in the DEN-treated *LIrs1KO* mice, whereas the tumor number and sizes were similar between the DEN-treated *LIrs2KO* mice and DEN-treated control mice (Fig. 2D,E). The body weight, blood glucose levels and serum insulin levels, which could also affect the risk of hepatocarcinogenesis, were also examined at 10 months after the DEN administration; although the slight increase in body weight and relatively high glucose and insulin levels in the *LIrs1KO* mice seemed to reflect the better nutritional status of these mice due to the suppressed hepatocarcinogenesis, these parameters in the *LIrs2KO* mice were comparable to those in the control mice (Supplementary Figure S3A,B,C). There is the possibility that the hepatocytes which escaped gene ablation had a survival advantage and developed into HCC^19^. However, in this study, the expression levels of Irs1 or Irs2 was still ablated in the tumors from *LIrs1KO* mice and *LIrs2KO* mice, respectively (Supplementary Figure S4).

Since it was possible that knockout of Irs could have affected the initial hepatic response to DEN, the short term response was assessed 48 h after single administration of DEN (100 mg/kg) in adult mice. There were no differences in the serum ALT levels either between *LIrs1KO* and control mice or between *LIrs2KO* and control mice (Supplementary Figure S5A). The expression levels of Bcl-XL (anti-apoptosis molecule) and Bax (pro-apoptosis molecule) following acute DEN exposure were also similar between the *LIrs1KO* and control mice as well as *LIrs2KO* and control mice (Supplementary Figure S5B). These data suggest that deletion of Irs1, but not of Irs2, in the hepatocytes suppressed the development of DEN-induced HCC.

### Loss of Irs1 in the hepatocytes decelerates tumor growth and proliferation

**Figure 3:**
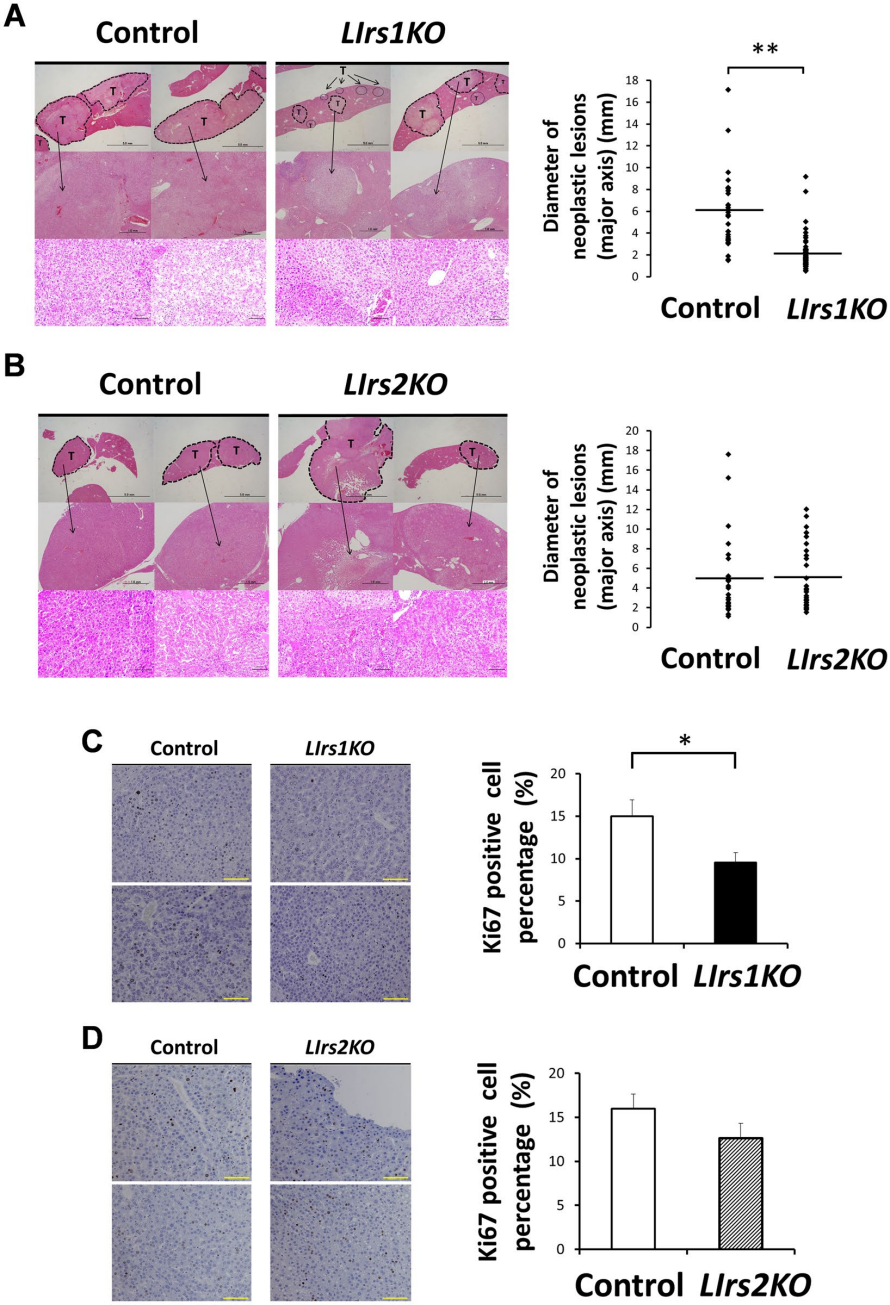
Loss of Irs1 in the hepatocytes decelerates tumor growth and proliferation. Ten months after DEN administration, histological analyses were performed. (**A** and **B**) Representative images of hematoxylin and eosin (H&E) staining (left) and diameter of neoplastic lesions (mm) (major axis)/field (right) in the livers from each DEN-treated mouse group (11–16 mice in each group). T, tumor lesion. Scale bar, 5 mm, 1 mm, 100 μm, in order from the top. (**C** and **D**) Representative images of Ki67 immunostaining (left) and the percentage of Ki67 positive cells in neoplastic lesions in the livers from each DEN-treated mouse group (right). Scale bar, 100 μm. Values are the means ± SEM of data obtained from the analysis of each group (n = 11–12). *P *<* 0.05. **P *<* 0.01.

Histological analysis using hematoxylin and eosin (H&E) staining demonstrated that the HCCs in each group of mice were relatively well-differentiated tumors. There were no significant differences in the degree of tumor differentiation among the groups of mice (Fig. 3A,B, left panels). Although microscopic examination revealed several tiny tumors in the DEN-treated *LIrs1KO* mice that were not detected on macroscopic examination, the sizes of the neoplastic lesions were still significantly smaller in these mice than those in the DEN-treated control mice (Fig. 3A, right panel). On the other hand, the sizes of the neoplastic lesions as assessed by microscopic examination were similar between the DEN-treated *LIrs2KO* mice and DEN-treated control mice (Fig. 3B, right panel), consistent with the findings on macroscopic examination. In the absence of DEN administration, both *LIrs1KO* mice and *LIrs2KO* mice exhibited only low counts of Ki67-positive proliferating cells, with no significant differences as compared to the counts in the corresponding control mice (Supplementary Figure S6); these results suggest that Irs1 or Irs2 *per se* did not affect the hepatocyte proliferative activity in the normal liver. After the development of HCC induced by DEN, however, the percentage of Ki67-positive proliferating cells in the tumor area were significantly lower in the DEN-treated *LIrs1KO* mice (Fig. 3C), but not in the DEN-treated *LIrs2KO* mice (Fig. 3D). These results suggest that ablation of Irs1 suppressed the proliferation of tumor cells, decelerating growth of HCC.

### Tumors from DEN-treated *LIrs1KO* mice exhibit decreased activation of Akt, along with the decreased expression levels of genes related to inflammation and invasion

**Figure 4:**
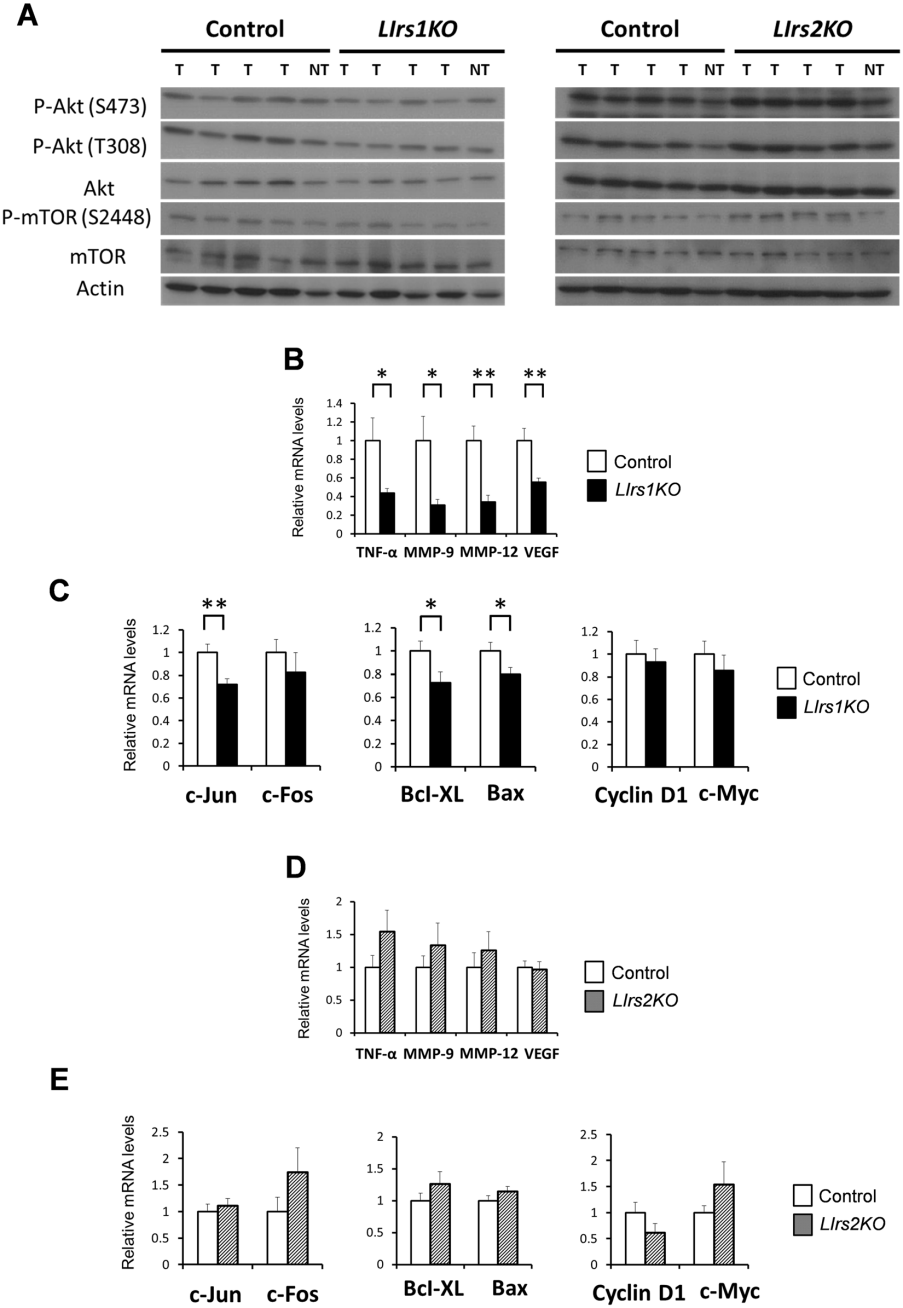
Tumors from DEN-treated *LIrs1KO* mice exhibit decreased activation of Akt, along with the decreased expression levels of genes related to inflammation and invasion. (**A**) Phosphorylation and protein expression levels of Akt and mTOR. Lysates from tumors (T) and non-tumor tissues (NT) in each DEN-treated mouse group were immunoblotted with the indicated antibodies. (**B**–**E**) RT-PCR analysis of indicated genes in tumors from DEN-treated *LIrs1KO* and DEN-treated control mice (**B** and **C**), or DEN-treated *LIrs2KO* and DEN-treated control mice (**D** and **E**). **B** and **C**, n = 10–12; **D** and **E**, n = 7–8. Values are the means ± SEM of data obtained from the analysis of each group. *P *<* 0.05. **P *<* 0.01.

Why do *LIrs1KO*, but not *LIrs2KO*, mice exhibit suppressed DEN-induced HCC development? Immunoblot analysis revealed decreased protein and phosphorylation levels of Akt as well as low phosphorylation levels of mTOR in the tumors in the DEN-treated *LIrs1KO* mice as compared to those in the DEN-treated control mice (Fig. 4A, left panel). In contrast, these differences were not observed between the DEN-treated *LIrs2KO* mice and DEN-treated control mice (Fig. 4A, right panel). To better understand the characteristics of the tumors lacking in Irs1, we examined the expressions of various genes related to HCC progression in the tumors from each group of mice. Expression levels of TNF-α, MMP-9, MMP-12, VEGF, which are elevated in HCC, were significantly lower in the tumors from the DEN-treated *LIrs1KO* mice than in the DEN-treated control mice (Fig. 4B), suggesting that tumors lacking in Irs1 may have less inflammatory and invasive potential. The expression level of c-Jun, but not of c-Fos, was significantly lower in the tumors from the DEN-treated *LIrs1KO* (Fig. 4C). Furthermore, expression levels of both Bcl-XL and Bax were significantly lower in the tumors from the DEN-treated *LIrs1KO* mice (Fig. 4C). On the other hand, there were no significant differences in the tumor expression levels of any of these genes between the DEN-treated *LIrs2KO* and DEN-treated control mice (Fig. 4D,E). The tumor expression levels of cyclin D1 and c-Myc, which are major target genes of the Wnt/β-catenin signaling pathway, were comparable between the DEN-treated *LIrs1KO* and DEN-treated control mice, as well as between the DEN-treated *LIrs2KO* and DEN-treated control mice (Fig. 4C,E). These data suggest that insulin signaling in HCC, mainly mediated by Irs1, and not Irs2, is associated with inflammation and invasion during hepatocarcinogenesis.

### Irs1 is regulated by Wnt/β-catenin signaling in the hepatocytes

**Figure 5:**
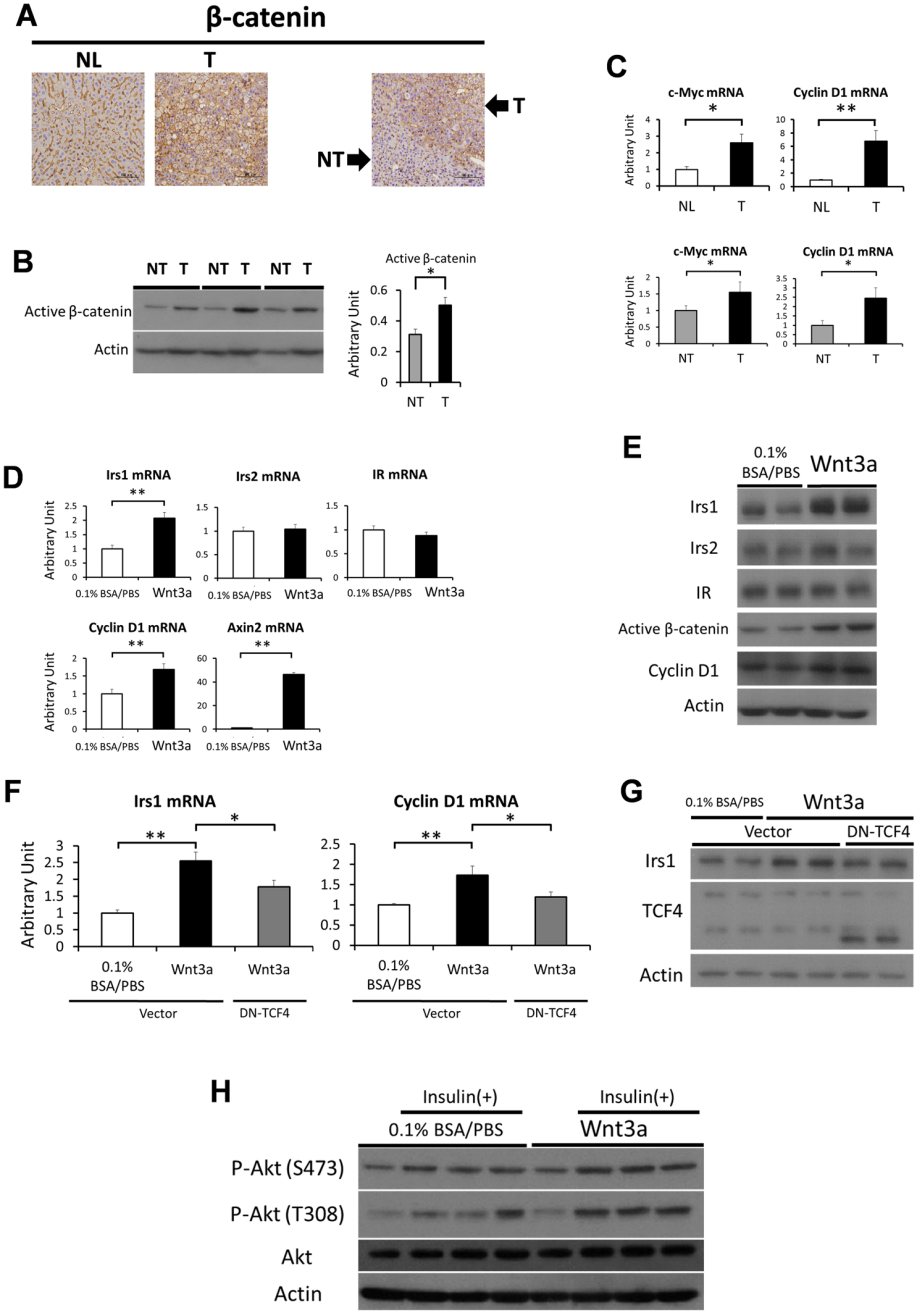
Irs1 is regulated by Wnt/β-catenin signaling in the hepatocytes. (**A**) Immunostaining of β-catenin in normal liver (NL) and tumor (T), or tumor (T) and matched non-tumor tissue (NT). Scale bar, 100 μm. (**B**) Protein expression levels of active (dephosphorylated on Ser37 or Thr41) β-catenin in tumors and matched non-tumor tissues. Representative immunoblot analysis for active β-catenin (left panel). Results were quantified and normalized to Actin (right panel, n = 6, paired t test). (**C**) Expression levels of Wnt target genes in tumors (T) and normal livers (NL) (upper panel, n = 7–8), or tumors (T) and matched non-tumor tissues (NT) (lower panel, n = 7, paired t test). (**D**) Expression levels of Irs1, Irs2, IR, cyclin D1 and Axin2 genes after treatment for 48 h with 50 ng/ml of recombinant Wnt3a in mouse primary hepatocytes (n = 6 per condition) (**E**) Lysates from primary hepatocytes treated with 50 ng/ml of recombinant Wnt3a for 48 h were immunoblotted with the indicated antibodies. (**F** and **G**) Hepatocytes were transfected with dominant-negative TCF4 cDNA (DN-TCF4) or control vector for 4 h. Thereafter, they were stimulated for 44 h by low serum medium with or without 50 ng/ml mouse recombinant Wnt3a. (**F**) Expression levels of Irs1 and cyclin D1 genes (n = 10 per condition), and (**G**) protein levels of Irs1, TCF4. (**H**) Phosphorylation levels of Akt after Wnt3a and insulin stimulation. After primary hepatocytes were incubated for 48 h by low serum medium with or without 50 ng/ml mouse recombinant Wnt3a, they were stimulated with 100 nM insulin for 30 min. Lysates were immunoblotted with the indicated antibodies. Values are the means ± SEM of data obtained from the analysis of each group. *P *<* 0.05. **P *<* 0.01.

To evaluate the mechanism of upregulation of Irs1 in HCC, we focused on Wnt/β-catenin signaling because recent studies have reported that this signaling regulates the expression of Irs1 in other tissues^20^. Ten months after administration of DEN or normal saline to wild-type mice, immunohistochemical analysis revealed increased β-catenin expression in the tumor lesions as compared to the normal liver and non-tumor tissues (Fig. 5A). The expression levels of active β-catenin were significantly higher in tumors as compared to matched non-tumor tissues (Fig. 5B). Major target genes of Wnt/β-catenin signaling were also upregulated in the tumors (Fig. 5C). These results suggest that Wnt/β-catenin signaling was enhanced in these tumors. To assess regulation of Irs1 by Wnt/β-catenin signaling, primary hepatocytes from wild type mice (C57BL/6 J) were stimulated with mouse recombinant Wnt3a, which can activate canonical Wnt/β-catenin signaling. When target genes of Wnt/β-catenin signaling such as cyclin D1 and Axin2, were upregulated, expression of Irs1, but not that of Irs2 or IR, was significantly elevated (Fig. 5D). Irs1 protein expression was also increased by Wnt3a treatment (Fig. 5E). Because TCF4 (TCF7L2) is one of the major Wnt/β-catenin signaling-associated transcription factors and binds to Irs1 promotor in the rat hepatoma cell line H4IIE^16, 21^, the effect of dominant-negative TCF4 cDNA (DN-TCF4) on the regulation of Irs1 in primary hepatocytes was examined. Transient transfection of DN-TCF4 reduced Irs1 induction by Wnt3a at both the mRNA and protein levels (Fig. 5F,G). Wnt3a treatment also increased insulin-stimulated phosphorylation levels of Akt (Fig. 5H). These data suggest that Irs1 was a target gene of the Wnt/β-catenin signaling pathway in the hepatocytes, associated with enhanced insulin signaling. Moreover, it seemed reasonable to consider that Irs1 upregulation in the HCC tumors was caused by activated Wnt/β-catenin signaling.

### Irs1 appears to be required for promotion of the Warburg effect and change in fat metabolism during hepatocarcinogenesis

**Figure 6:**
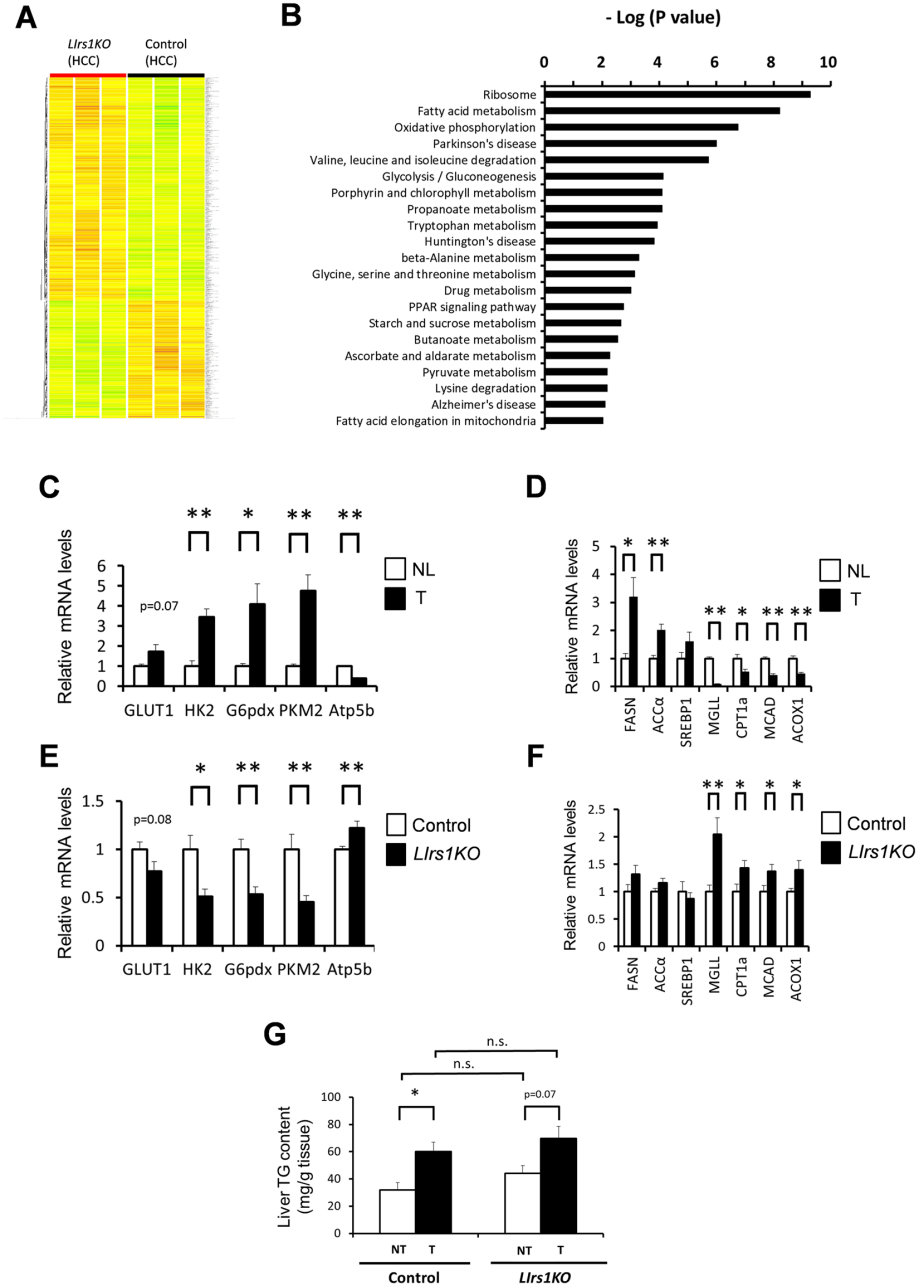
Irs1 appears to be required for promotion of the Warburg effect and change in fat metabolism during hepatocarcinogenesis. (**A**) Microarray analysis and clustering of genes in tumors from DEN-treated *LIrs1KO* and DEN-treated control mice. The Heatmap showed all genes changed with p < 0.05. Red, upregulated; green, downregulated; yellow, no change (n = 3). (**B**) KEGG pathway analysis was performed with the program DAVID. Pathways changed with p < 0.01 were listed. (**C** and **D**) RT- PCR analysis of indicated gene expression levels related to enzymes of (**C**) glucose metabolism and (**D**) fat metabolism in tumors (T) and normal livers (NL) (n = 5). (**E** and **F**) RT- PCR analysis of indicated gene expression levels related to enzymes of (E) glucose metabolism and (**F**) fat metabolism in tumors from DEN-treated *LIrs1KO* and DEN-treated control mice (n = 10–12). (**G**) The TG content of the tumors (T) and non-tumor tissues (NT) in DEN-treated *LIrs1KO* and DEN-treated control mice (n = 6). Values are the means ± SEM of data obtained from the analysis of each group. *P *<* 0.05. **P *<* 0.01. (n.s., not significant difference.)

For a more comprehensive understanding of the characteristics of tumors lacking in Irs1, we conducted gene expression microarray analysis to compare the tumor expression profiles of genes in the DEN-treated *LIrs1KO* mice as compared to the DEN-treated control mice. Expressions of approximately 4000 genes were significantly altered (p < 0.05) (Fig. 6A, Supplementary Table S1). KEGG (Kyoto Encyclopedia of Genes and Genomes) pathway analyses using DAVID indicated that the genes involved in metabolic pathways, including those of protein, glucose and fat were also altered (Fig. 6B). The differences in the ribosome pathway presumably reflected the low level of activation of mTOR signaling in the tumors from the DEN-treated *LIrs1KO* mice, as borne out by the decreased phosphorylation of mTOR (Fig. 4A, left panel). To determine glucose and fat metabolism in HCC, we again compared DEN-induced HCC with normal liver tissues at 10 months after administration of DEN or normal saline using wild type mice (C57BL/6 J). Elevated expression levels of the genes encoding glucose transporter 1 (GLUT1), hexokinase-2 (HK2), glucose-6-phosphate dehydrogenase (G6pdx), pyruvate kinase M2 (PKM2), and reduced expression levels of ATP synthase subunit beta (Atp5b) were observed in the tumors (Fig. 6C), which is well-known as the Warburg effect. In regard to fat metabolism, while the expression levels of genes related to lipogenesis (FASN; fatty acid synthase, ACCα; acetyl-CoA carboxylase α) were upregulated in the tumors (Fig. 6D), those of genes related to lipolysis (MGLL; monoacylglycerol lipase) and β-oxidation (CPT1a; carnitine palmitoyltransferase 1a, MCAD; medium-chain acyl-CoA dehydrogenase, ACOX1; acyl-CoA oxidase 1) were downregulated in the tumors (Fig. 6D). Although fat metabolism could be affected by the nutritional status of individual, comparing tumors with matched non-tumor tissues in each individual revealed the tendency towards upregulated expression of lipogenic enzyme genes and significantly downregulated expressions of genes related to β-oxidation in the tumor lesions (Supplementary Figure S7). We observed lower tumor expression levels of GLUT1, HK2, G6pdx and PKM2, and higher expression levels of Atp5b gene in the DEN-treated *LIrs1KO* mice as compared to the DEN-treated control mice, suggesting that the Warburg effect was suppressed in the tumors from the DEN-treated *LIrs1KO* mice (Fig. 6E). While we did not find any difference in the expression levels of genes encoding lipogenic enzymes, the expression levels of genes involved in lipolysis and β-oxidation were significantly higher in the tumors from DEN-treated *LIrs1KO* mice as compared to the DEN-treated control mice (Fig. 6F). Although the TG contents of the tumors were elevated because of increased lipogenesis and decreased lipolysis and β-oxidation, the TG contents of the tumors and non-tumor tissues were similar in the DEN-treated *LIrs1KO* and DEN-treated control mice (Fig. 6G). These data suggest that insulin signaling mediated by Irs1 in HCC is associated with promotion of the Warburg effect and alterations of fat metabolism during hepatocarcinogenesis.

## Discussion

**Figure 7:**
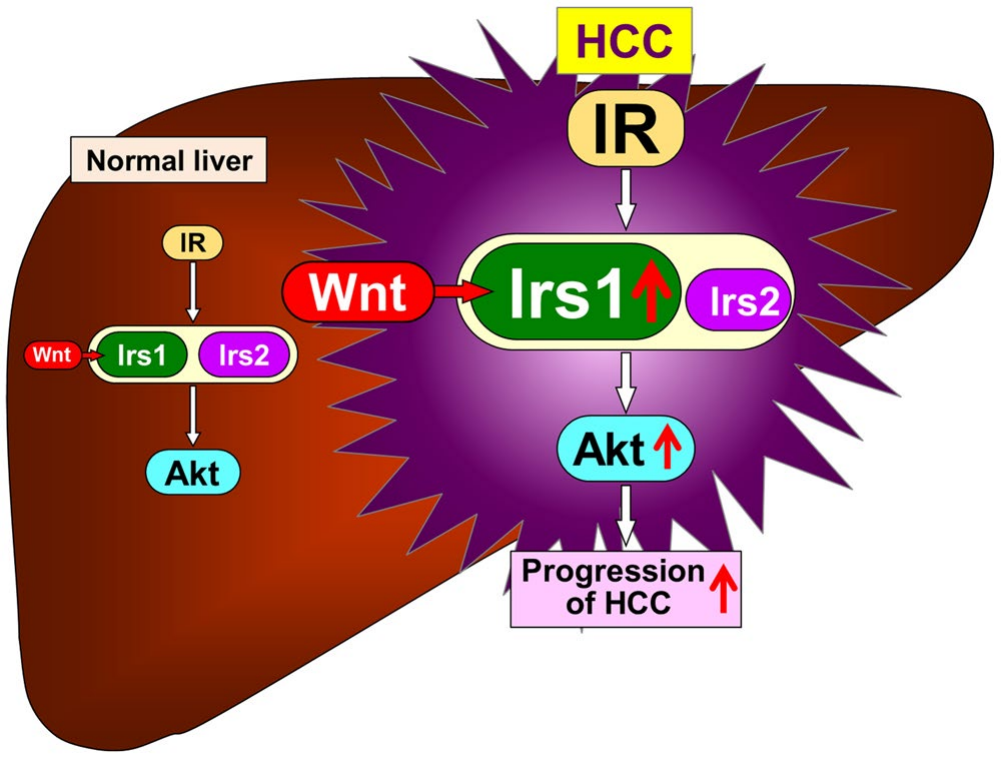
Schematic representation of mechanisms underlying crosstalk between Wnt/β-catenin signaling and insulin signaling via Irs1 during hepatocarcinogenesis. Wnt/β-catenin signaling is activated during hepatocarcinogenesis due to somatic mutations of various genes related to Wnt/β-catenin signaling. Activated Wnt/β-catenin signaling upregulates Irs1 gene and protein expression. Upregulated Irs1, but not Irs2, enhances insulin signaling including Akt pathway and promotes the progression of HCC.

While Irs1 and Irs2 exhibit high structural homology and are abundantly expressed at similar levels in the normal liver^22–25^, Irs1 expression was markedly upregulated in HCC as compared to Irs2 (Fig. 1A,B, Supplementary Figure S1), as previously reported^9^. Although the precise mechanism underlying the slight upregulation of Irs2 in HCC remains unclear, it was reported recently that upregulation of Irs1 expression in other tissues was mediated by Wnt/β-catenin signaling^20, 26^. In fact, Irs1, but not Irs2, mRNA and protein expression levels in the hepatocytes were significantly increased by Wnt3a, an activator of canonical Wnt/β-catenin signaling (Fig. 5D,E). Dysregulation of Wnt/β-catenin signaling is a major event in the development of HCC in humans^27^ and mice^28^. On the basis of these findings, we propose that the increased level of β-catenin in HCC, which is observed in approximately 50–70% of HCC^29^, provides a growth advantage to tumor cells by promoting their proliferation via upregulation of Irs1. In the normal state, β-catenin is phosphorylated by glycogen synthase kinase-3β and degraded by the ubiquitin-proteasome pathway. On the other hand, in HCC, once accumulation of β-catenin occurs as a result of somatic mutations within the APC or β-catenin gene, not only genes involved in cell proliferation, such as c-Myc and cyclin D1, but also the Irs1 genes are upregulated, which activates insulin signaling including Akt phosphorylation, and further stimulates proliferation of HCC. Consistent with this, DEN-induced hepatocarcinogenesis was dramatically suppressed in the livers of *LIrs1KO* mice. Moreover, in these tumors, the expression levels of the genes involved in the Warburg effect, inflammation and invasion were also suppressed as compared with those in the control mice. These data suggest that upregulated Irs1 by Wnt/β-catenin signaling is a predominant mediator of insulin signaling in HCC, and is also associated with promotion of the progression of HCC (Fig. 7).

It is apparent that some interplay exists between increased insulin/IGF1 signaling and HCC development, although the precise nature of the interplay remains elusive^3^. Although previous studies have shown the role of insulin/IGF1 signaling and the downstream molecules in HCC development, the possibility of the effect of steatosis and surrounding hepatocellular damage could not be excluded in these studies. In hepatocyte-specific PTEN (phosphatase and tensin homolog)- knockout mice, which exhibit hypersensitivity to insulin because of a lack of the negative regulator PTEN of insulin signaling, HCC development was significantly enhanced, accompanied by massive hepatic steatosis with hepatomegaly and steatohepatitis^30^. Conversely, a protective effect against liver tumors, including hepatic adenoma, induced by long-term high fat diet has been reported in systemic Irs1 knockout mice, with improvement of the steatosis^31^. In the present study, we demonstrated that insulin and/or IGF1 signaling mediated through Irs1 is directly contributed to the development of HCC, independent of the development of steatosis. The hepatic TG content in the *LIrs1KO* mice was essentially equivalent to that in the control mice reared normal diet (Fig. 6G). These findings suggest that enhanced insulin and/or IGF1 signaling in the liver exacerbates the development/progression of HCC, independent of any metabolic disorder.

We considered that insulin signaling was enhanced in DEN-induced HCC, based on the result of increased IR protein expression (Fig. 1D) and emergence of the IR-A alternative splicing isoform related to high ligand sensitivity (Fig. 1E), and the elevated phosphorylation levels of Akt (Fig. 1C). Consistently, increased IR expression and emergence of the IR-A alternative splicing isoform has been demonstrated even in human HCC^32^. On the other hand, it is controversial as to whether the phosphorylation level of Akt is upregulated or not in HCC. Heterogeneous expression profiles of phosphorylated Akt have been found in human HCC^33^. Moreover, transcriptome analysis has revealed that a specific subgroup of cases with HCC exhibited marked activation of the Akt pathway, while other subgroups showed various levels of Akt activation^34^. Nevertheless, our data suggests that the phosphorylation levels of Akt were upregulated, when we collected tumors based on the macroscopic appearance and analyzed tumors as a whole. Furthermore, our results identified that tumors lacking in Irs1 showed lower levels of activation of the Akt pathway, along with decreased proliferative, inflammatory and invasive potential, whereas tumors lacking in Irs2 showed similar Akt phosphorylation levels to those in the controls. These data suggest that Irs1-mediated Akt phosphorylation is critical for HCC development.

Gene expression microarray and pathway analyses have also revealed that promotion of the Warburg effect and change in fat metabolism during hepatocarcinogenesis were suppressed in tumors lacking in Irs1. The Warburg effect refers to aerobic glycolysis, widely known as a hallmark of cancer metabolism^35^. Although HiF1α activated by hypoxia is the master regulator of the Warburg effect, insulin signaling also seems to contribute to the Warburg effect via stabilization of the HIF1 complex^36^. Other enzymes related to the Warburg effect have also been reported to be regulated by insulin signaling, such as PKM2^37^ and G6pdx^38^. Although we speculate that insulin signaling via Irs1 might directly regulate the emergence of the Warburg effect during hepatocarcinogenesis, there still remains the possibility of an indirect effect, which needs further investigation.

Fatty acid synthesis is elevated in various cancers including human HCC^39^. On the other hand, the role of lipolysis and β-oxidation in HCC is controversial. While some studies have reported elevated β-oxidation in HCC^40, 41^, other studies have shown reduction of fatty acid oxidation in HCC^42^, especially HCC with dedifferentiation^43, 44^. A recent study demonstrated that reduction of fatty acid oxidation through suppression of medium and long-chain acyl-CoA dehydrogenases by activated HIF1 enhanced the proliferation of cancer cells because of decreased reactive oxygen species levels^45^. The contradictory results from previous studies regarding lipolysis and β-oxidation in HCC are possibly attributable to the various backgrounds of the HCC, such as viral infection or NASH, or different time-courses of hepatocarcinogenesis. It remains unclear whether alterations of lipid metabolism in HCC are directly regulated by insulin signaling via Irs1 or not.

Taken together, in the present study, we demonstrated that insulin signaling was activated accompanied by increased Irs1 expression in DEN-induced HCC. Deletion of Irs1 significantly suppressed HCC development and progression, associated with decrease in cellular proliferation, inflammation and aerobic glycolysis, known as the Warburg effect. Irs1 inhibition, such as degradation of Irs1 through promotion of Ser-phosphorylation^46^, may represent a potential therapeutic strategy for HCC.

## Materials and Methods

### Animals and treatments

C57BL/6 J mice were purchased from CLEA Japan. Generation of liver-specific Irs1-knockout (*LIrs1KO*) mice (*AlbCreIrs1* ^*lox/lox*^) and liver-specific Irs2-knockout (*LIrs2KO*) mice (*AlbCreIrs2* ^*lox/lox*^) has been described previously^7^. *Irs1* ^*lox/lox*^ and *Irs2* ^*lox/lox*^ mice were used as the controls. *LIrs1KO* mice, *LIrs2KO* mice, and their respective control mice were maintained on a C57/BL6 plus 129 Sv mixed genetic background. The mice were housed under a 12-h light-dark cycle and given access *ad libitum* to regular chow, CE-2 (CLEA Japan), consisting of 24.9% (w/w) protein, 4.1% fiber, 6.6% ash, 51.0% carbohydrates, 4.6% fat, and 8.9% water. All animal care and experiments were performed in accordance with the principles of the “Guide to the Care and Use of Experimental Animals” and were approved by the Animal Care Committee of the University of Tokyo. For establishing the mouse model of HCC for this study, 15-day-old male mice were injected intraperitoneally with 25 mg/kg of diethylnitrosamine (DEN, Sigma), and then weaned and maintained on standard chow. After 10 months, the mice were sacrificed for histological and biochemical analyses. In addition, for short term studies of DEN-induced hepatic injury, 12-week-old male mice were injected intraperitoneally with 100 mg/kg of DEN, and sacrificed 48 h later. In all the experiments, the mice were denied access to food overnight before they were sacrificed.

### Liver Tumor Analysis

Immediately after the mice were sacrificed, the livers were removed, washed in cold PBS and weighed. The number of visible surface liver tumor nodules that were more than 1mm in diameter was counted macroscopically. The maximal tumor size was determined by measuring the diameter of the major axis of each surface liver tumor nodule.

### Hepatocyte isolation and treatments

Hepatocytes were isolated from 8 to12-week-old C57BL/6 J mice by collagenase perfusion. After the mice were anesthetized with pentobarbital, their livers were perfused via the portal vein for 5 minutes (total volume of 20–25 ml) with Ca^2+^-free solution (136 mM NaCl, 5 mM KCl, 0.5 mM NaH_2_PO_4_ · 2H_2_O, 0.42 mM Na_2_HPO_4_ · 12H_2_O, 4.1 mM NaHCO_3_, 5 mM D-glucose, 0.5 mM EGTA, 10 mM HEPES, pH 7.4), followed by continuous perfusion with 40 ml of Hanks’ Balanced Salt solution (Sigma-Aldrich) containing 20 mg collagenase L (Wako Pure Chemical Industries Ltd) and 20 mg DISPASE®II (Wako Pure Chemical Industries Ltd), and 25 mM HEPES. About 15 min after the collagenase perfusion, livers were removed from the mice and dissociated in ice-cold wash buffer (William’s medium E containing 10% FBS) and filtered. The hepatocytes were washed thrice with ice-cold wash buffer by centrifugation at 50 x g for 3 min. The cells were plated on collagen-coated dishes in William´s medium E supplemented with 15% FBS, 100 nM dexamethasone, 100 nM insulin, 1% GlutaMAX™ (Thermo Fisher Scientific Inc.) and 1% penicillin/streptomycin solution. The hepatocytes were incubated in a humidified atmosphere at 37 °C and 5% CO2. Unattached cells were removed six hours after plating by medium exchange. One day after the plating, the medium was replaced by low serum medium (William’s medium E supplemented with 1% FBS, 1% GlutaMAX™ and 1% penicillin/streptomycin solution) with or without 50 ng/ml of mouse recombinant Wnt3a (R&D Systems) for stimulating canonical Wnt/β-catenin signaling. PBS containing 0.1% bovine serum albumin (0.1% BSA/PBS) was used as the control treatment.

### Transient plasmid transfections

Dominant-negative TCF4 (*TCF7L2*) cDNA is described elsewhere^47^. The empty pcDNA3.1 ( + ) vector was used as a negative control. Hepatocytes were transfected with each plasmid using Lipofectamine 2000 (Invitrogen) according to the manufacturer’s protocol.

### Histology and Immunostaining

Liver tissue was fixed overnight in 10% neutralized formaldehyde, and then dehydrated and embedded in paraffin. Sections were cut at 2 μm and stained with hematoxylin and eosin (H&E), and histologically evaluated. Immunohistochemical analysis was performed using anti-Ki67 antibody (Cell Signaling Technology) or anti-β-Catenin antibody (BD Biosciences). DAPI was used to stain the nuclei. Ki67 positive cell percentage was calculated by dividing the number of Ki67 positive cells by the number of unstained cells.

### Immunoprecipitation, Western Blot Analysis

To prepare tissue lysates, frozen liver samples were homogenized in buffer A (25 mM Tris-HCl [pH 7.4], 10 mM sodium orthovanadate, 10 mM sodium pyrophosphate, 100 mM sodium fluoride, 10 mM EDTA, 10 mM EGTA) containing protease inhibitor cocktail and 1% NP-40. The antibodies used were anti-Irs1 (Millipore), anti-Irs2 (Santa Cruz) and anti-IR-β (Santa Cruz) for immunoprecipitation, and anti-Irs1 (Millipore), anti-Irs2 (Cell Signaling), anti-IR-β (Santa Cruz), anti-Akt (Cell Signaling), anti-phospho (S473)-Akt (Cell Signaling), anti-phospho (T308)-Akt (Cell Signaling), anti-active β-catenin (Millipore), anti-cyclin D1 (Santa Cruz), anti-phospho (S2448)-mTOR (Cell Signaling), anti-mTOR (Cell Signaling), anti-TCF4 (Cell Signaling) and anti-Actin (Santa Cruz) for immunoblotting. Immunoprecipitation was performed before immunoblotting to assess Irs1, Irs2 and IR-β protein expression in the liver samples from the mice. For immunoprecipitation, 1 mg of the liver extracts was incubated with specific antibodies for 3 hr at 4 °C. Thereafter, protein G-Sepharose was added, followed by incubation for 2 hr at 4 °C. After washing three times, the immunocomplexes were analyzed by Western blotting. Sample buffer for western blotting was composed of 3% SDS, 50 mM Tris-HCl (pH 6.8), 5% 2-mercaptoethanol, and 10% glycerol. Samples were mixed with 5 × sample buffer, heated at 95 °C for 5 min for heat denaturation, separated on polyacrylamide gels, and transferred to a Hybond-P polyvinylidene difluoride transfer membrane (Amersham Biosciences). Bands were detected with ECL detection reagents (Amersham Biosciences). Image J software was used to quantify the density and size of the blots, and values were normalized to Actin.

### Biochemical assays

Blood glucose was measured using an automatic glucometer (Sanwa Kagaku Kenkyusho Co., Ltd). Whole blood was collected and centrifuged, and the separated serum samples were stored at −80 °C. Serum insulin levels were determined using the mouse insulin ELISA kit (Morinaga, Japan). Serum alanine aminotransferase levels were measured using the Transaminase CII-test kit (Wako Pure Chemical Industries Ltd). For determining the TG content in the liver, the tissue homogenates were extracted with 2:1 (vol/vol) chloroform/methanol. Chloroform/methanol was added to the homogenate, and the mixture was shaken for 15 min. After centrifugation at 14,000 rpm for 10 min, the organic layer was collected. This extraction was repeated three times, and the collected sample was dried and resuspended in 1% Triton X-100/ethanol. The measurement was conducted using Triglyceride E-test Wako (Wako Pure Chemical Industries, Ltd., Osaka, Japan).

### Real-time quantitative PCR

Total RNA was extracted from various tissues with TRIzol reagent (Invitrogen) according to the manufacturer’s instructions. After treatment with RNase-Free DNase (Qiagen) to remove any residual contaminant DNA, cDNA was synthesized with MultiScribe Reverse-Transcriptase (Applied Biosystems, Foster City, CA). Real-time quantitative PCR (RT-PCR) was performed on an ABI Prism 7000 sequence detector equipped with a thermocycler, using Gene Expression Master Mix or SYBR Green PCR Master Mix (Applied Biosystems). The probes and primer sequences that were used are shown in Supplementary Table S2. The expression was normalized to the expression level of the housekeeping gene *cyclophilin*.

### Microarray analysis

Cyanine 3-labeled cRNA was prepared from 50 ng total RNA using the Low Input Quick Amp Labeling Kit (Agilent) according to the manufacturer’s instructions. After purification, a total of 600 ng of cyanine 3-labeled cRNA was used for each hybridization. The Agilent Technologies Microarray Scanner was used for array scanning. The scanned images were analyzed with Agilent Feature Extraction 10.7.3.1. Raw data processing and normalization were performed using the GeneSpring GX, followed by t-test and clustering. Annotation enrichment and pathway analysis were performed using the DAVID (http://david.abcc.ncifcrf.gov/) web tools (version 6.7).

### Statistical analysis

The results are presented as mean ± standard error of the mean (SEM). The statistical significance was calculated by the unpaired Student’s t test, unless otherwise specified. The paired t test was performed in samples from the same individual. For experiments involving multiple comparisons, data were analyzed by the Tukey-Kramer test. P values of less than 0.05 were considered to indicate statistical significance.

## Electronic supplementary material

Supplementary Information

Supplementary Table S1
