# Supplementary material for: Role of insulin receptor substrates in the progression of hepatocellular carcinoma
Source: Sci Rep. 2017 Jul 14;7:5387. doi: 10.1038/s41598-017-03299-3 (PMC5511151; doi:10.1038/s41598-017-03299-3)
Supplement: Supplementary file 1 — Supplementary Information [file 41598_2017_3299_MOESM1_ESM.pdf]

SUPPLEMENTAL INFORMATION

Role of insulin receptor substrates in the progression of hepatocellular carcinoma

Yoshitaka Sakurai[1], Naoto Kubota[1,2,3,4]*, Iseki Takamoto[1], Atsushi Obata[5], Masahiko Iwamoto[1], Takanori Hayashi[1], Masakazu Aihara[1], Tetsuya Kubota[1,3,4], Hiroshi Nishihara[6,7], and Takashi Kadowaki[1]*

[1] Department of Diabetes and Metabolic Diseases, Graduate School of Medicine, The University of Tokyo, Tokyo, Japan

[2] Department of Clinical Nutrition Therapy, The University of Tokyo, Tokyo, Japan

[3] Clinical Nutrition Program, National Institute of Health and Nutrition, National Institutes of Biomedical Innovation, Health and Nutrition, Osaka, Japan

[4] Laboratory for Metabolic Homeostasis, RIKEN Center for Integrative Medical Sciences, Kanagawa, Japan

[5] Department of Diabetes, Endocrinology and Metabolism, Kawasaki Medical School, Kurashiki, Okayama, Japan

[6] Department of Translational Pathology, Hokkaido University Graduate School of Medicine, Sapporo, Hokkaido, Japan

[7] Translational Research Laboratory, Hokkaido University Hospital, Clinical Research and Medical Innovation Center, Sapporo, Hokkaido, Japan

Supplementary Figure S1

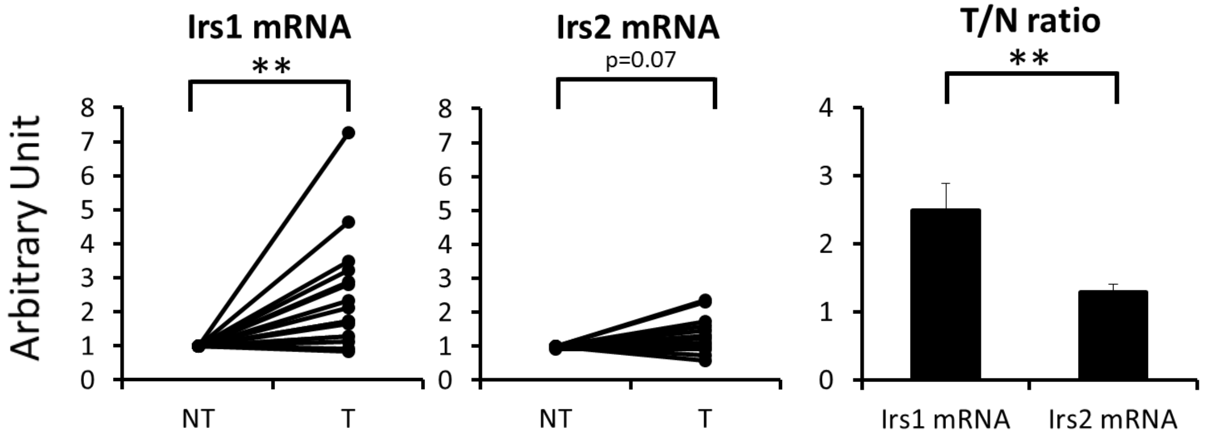

Supplementary Figure S1. Expression levels of Irs1 and Irs2 genes in the tumors (T) and matched non-tumor tissues (NT) in each individual mouse treated with DEN (n=17) (paired t test). The increase in the tumor: non-tumor tissue expression ratio for Irs1 mRNA was compared to that for Irs2 mRNA. ***P* < 0.01.

Supplementary Figure S2

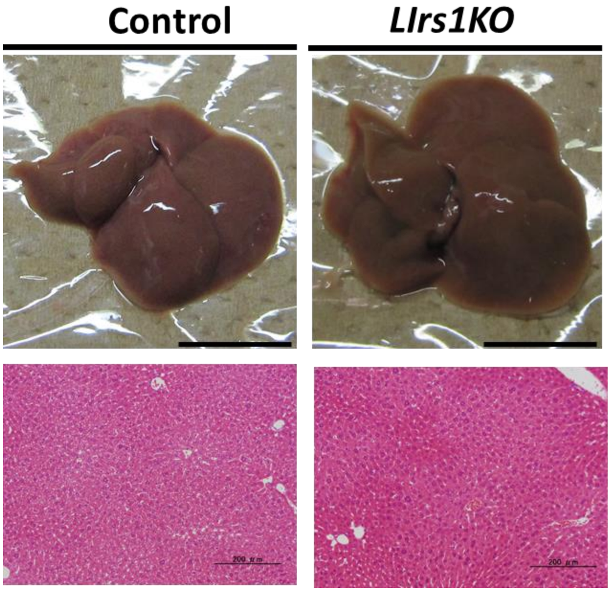

Supplementary Figure S2. Macroscopic and microscopic examination in the liver from each group of mice in the absence of DEN. Gross appearances and H&E staining of the representative livers in control (*Irs1*$^{lox/lox}$) and *LIrs1KO* mice, or control (*Irs2*$^{lox/lox}$) and *LIrs2KO* mice at 10 months after the normal saline administration. Scale bar, 10mm (gross appearances), 200 µm (H&E staining).

Supplementary Figure S3

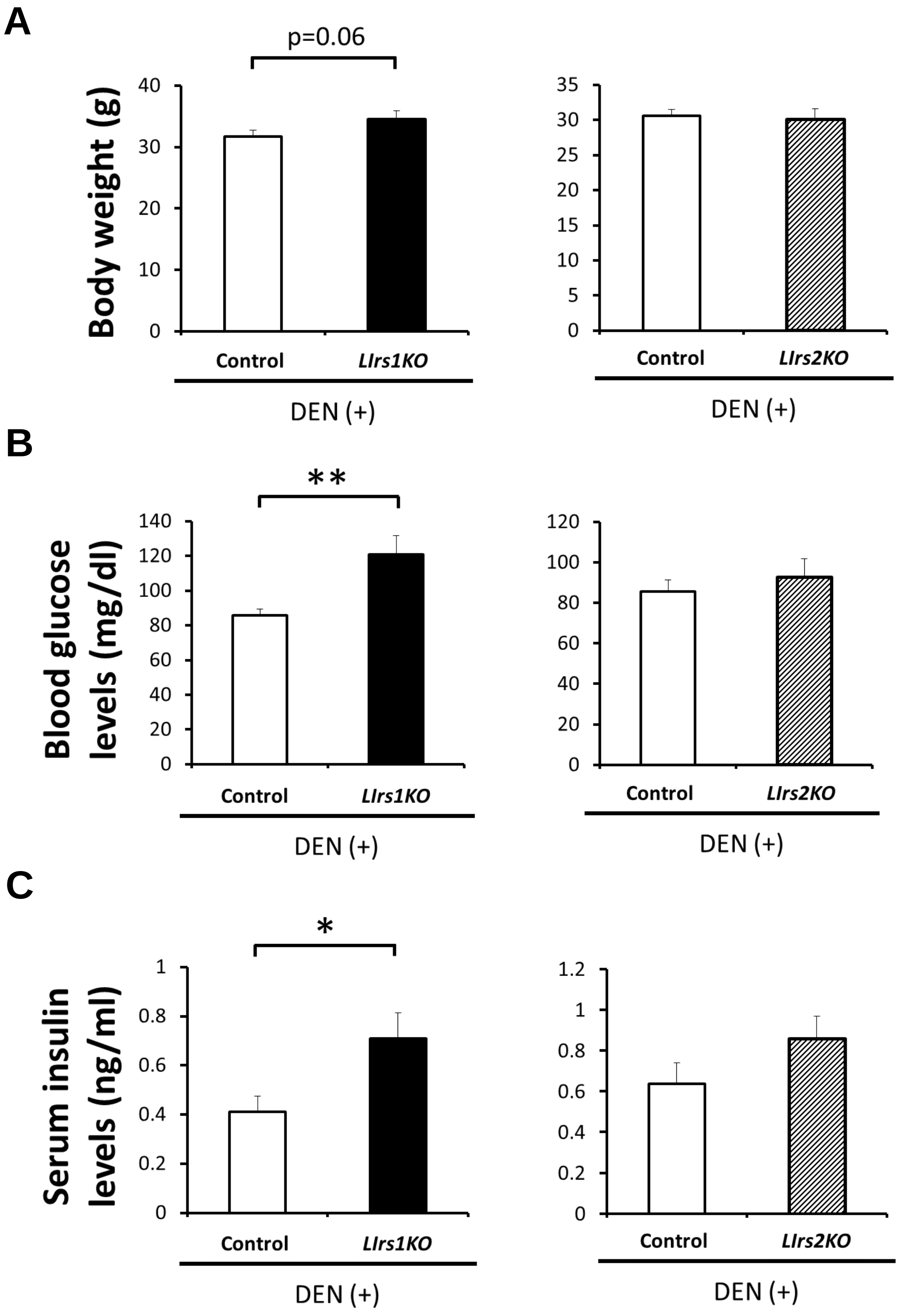

Supplementary Figure S3. Metabolic parameters at 10 months after DEN administration in each group of mice. (A) Body weight, (B) Fasting blood glucose levels, (C) Fasting serum insulin levels between 10-month-old DEN-treated control (*Irs1*$^{lox/lox}$) and *LIrs1KO* mice, or between 10-month-old DEN-treated control (*Irs2*$^{lox/lox}$) and *LIrs2KO* mice (n=11-12). *$P < 0.05$. **$P < 0.01$.

Supplementary Figure S4

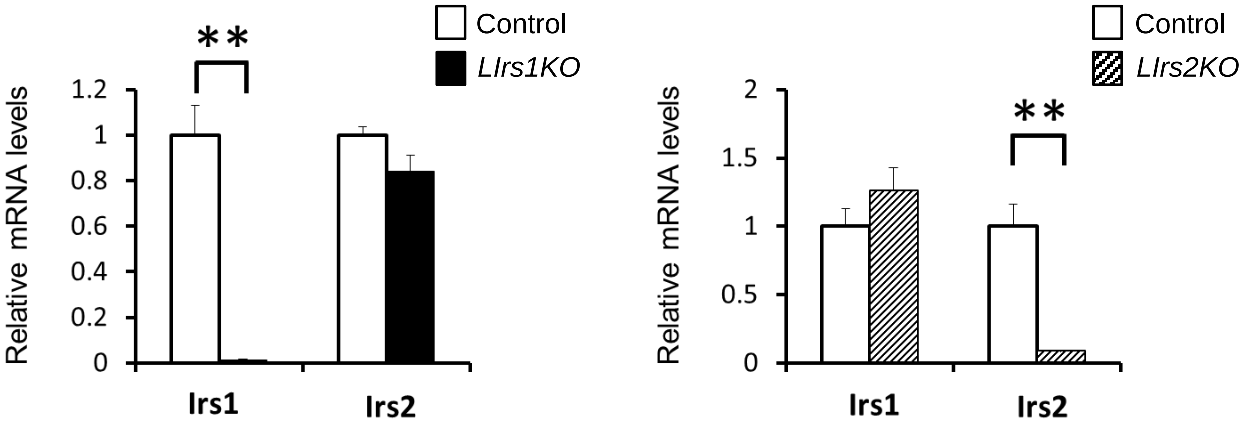

Supplementary Figure S4. Expression levels of Irs1 and Irs2 genes in tumors from each DEN-treated mouse group. Values are the means ± SEM of data obtained from the analysis of each group (n=8-11). ***P* < 0.01.

Supplementary Figure S5

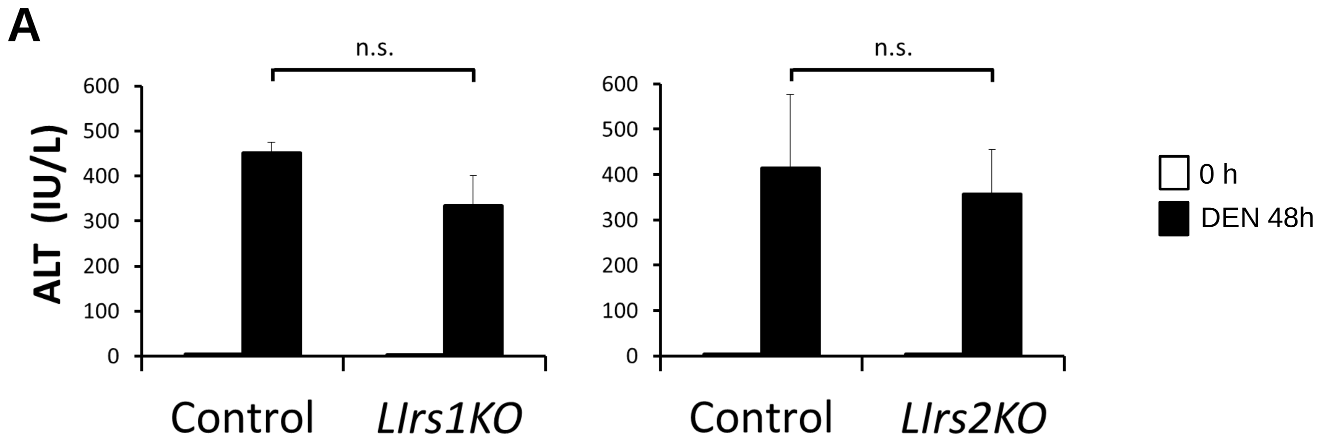

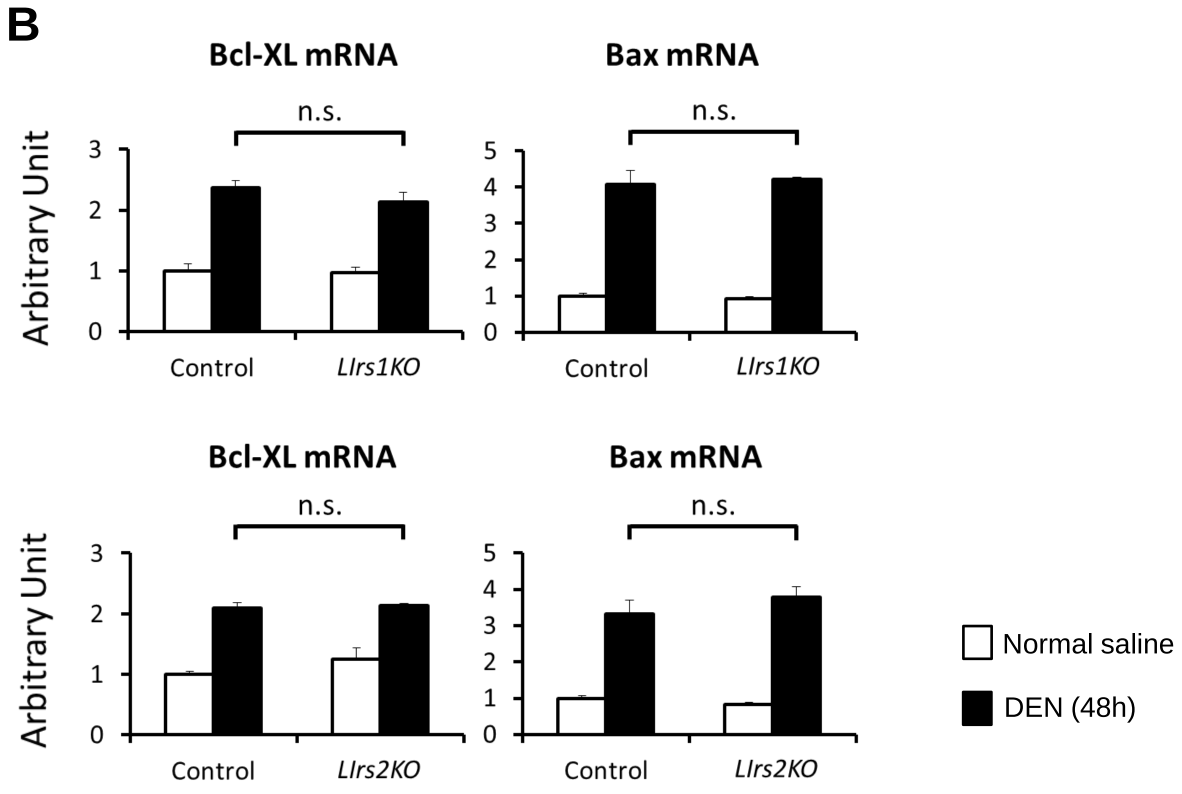

Supplementary Figure S5. Acute reaction in the liver following initial exposure of DEN. (A) Serum ALT levels (n=6-8) and (B) expression levels of Bcl-XL and Bax in the livers from control (*Irs1*$^{lox/lox}$) and *LIrs1KO* mice, or control (*Irs2*$^{lox/lox}$) and *LIrs2KO* mice at 48h after administration of DEN (100mg/kg) or normal saline (n=4). Values are the means ± SEM of data obtained from each group. (n.s., not significant difference.)

Supplementary Figure S6

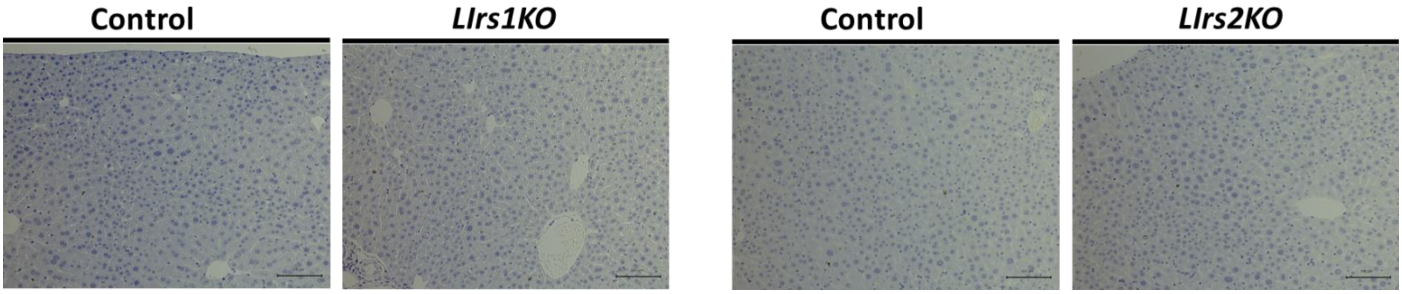

Supplementary Figure S6. Ki67 immunostaining in the liver from each group of mice in the absence of DEN. Representative images of Ki67 immunostaining in the livers from control (*Irs1*$^{lox/lox}$) and *LIrs1KO* mice, or control (*Irs2*$^{lox/lox}$) and *LIrs2KO* mice at 10 months after the normal saline administration. Scale bar, 100 μm.

Supplementary Figure S7

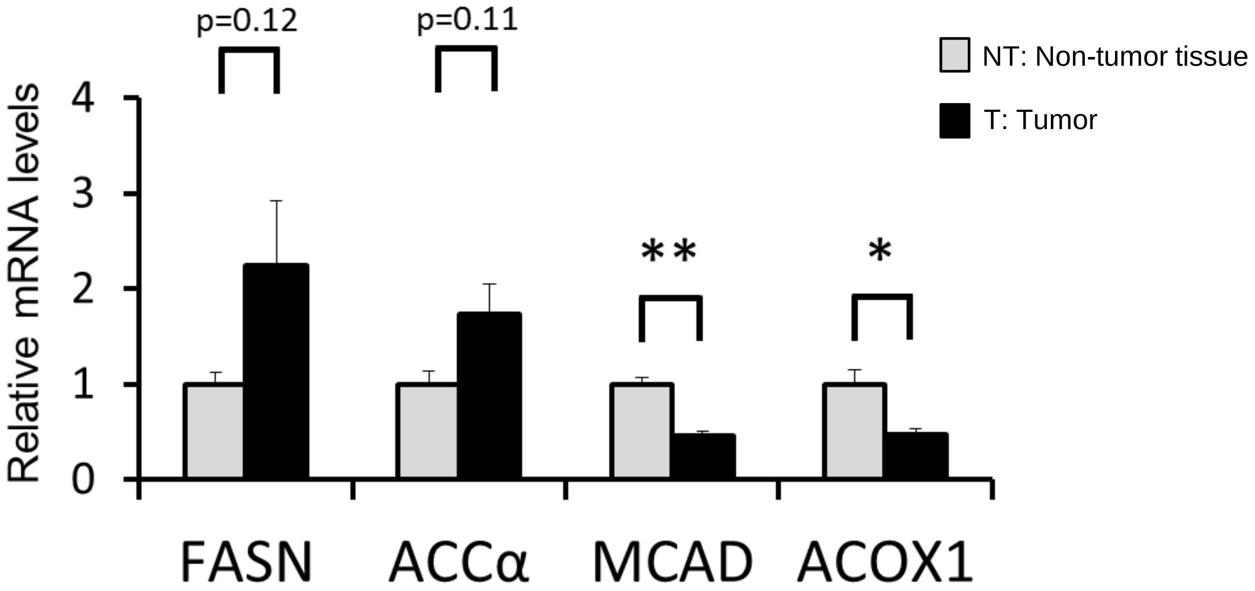

Supplementary Figure S7. Expression levels of lipogenic genes (FASN and ACCα) and genes related to β-oxidation (MCAD and ACOX1) in tumors (T) and matched non-tumor tissues (NT) in each individual mouse treated with DEN (C57BL/6J) (n=7) (paired t test). *$P < 0.05$. **$P < 0.01$.

Supplementary Table S2 List of probes and primer sequences for quantitative RT-PCR

TaqMan® probe

Irs1	Mm00439720_s1
Irs2	Mm03038438_m1
Axin2	Mm00443610_m1
Cyclophilin A	Mm02342429_g1

Primer sequences

AFP	Forward	5′-CACACCCGCTTCCCTCAT-3′
	Reverse	5′-TTTTCGTGCAATGCTTTGGA-3′
IR	Forward	5′-CCCGAGTGCCCGTCTGGCTA-3′
	Reverse	5′-GCCAGGTTGTTGCCCCCTCG-3′
IR (exon11)	Forward	5′-ATCAGAGTGAGTATGACGACTCGG-3′
	Reverse	5′-TCCTGACTTGTGGGCACAATGGTA-3′
GHR	Forward	5′-TTGTAGTTATATTTTCAAAGCAGCAAA-3′
	Reverse	5′-AGAAGATCTGGATCAATCCCTTT-3′
IGF1	Forward	5′-CTGGACCAGAGACCCTTTGC-3′
	Reverse	5′-GGACGGGGACTTCTGAGTCTT-3′
IGF2	Forward	5′-GTGCTGCATCGCTGCTTAC-3′
	Reverse	5′-ACGTCCCTCTCGGACTTGG-3′
IGF1R	Forward	5′-GTGGGGGCTCGTGTTTCTC-3′
	Reverse	5′-GATCACCGTGCAGTTTTCCA-3′
Cyclin D1	Forward	5′-GCGTACCCTGACACCAATCTC-3′
	Reverse	5′-CTCCTCTTCGCACTTCTGCTC-3′
c-Myc	Forward	5′-GACGAGCACAAGCTCACCTC-3′
	Reverse	5′-CCCCAGCCAAGGTTGTGAGG-3′
c-Jun	Forward	5′-CCGCCCCTGTCCCCTAT-3′
	Reverse	5′-TCCTCATGCGCTTCCTCTCT-3′
c-Fos	Forward	5′-CCCCAAACTTCGACCATGAT-3′
	Reverse	5′-GGAGGATGACGCCTCGTAGTC-3′
Bcl-XL	Forward	5′-GAATGGAGCCACTGGCCA-3′
	Reverse	5′-GCTGCCATGGGAATCACCT-3′
Bax	Forward	5′-CCAGGATGCGTCCACCAAGAA-3′
	Reverse	5′-CTCTGCAGCTCCATATTGCTGT-3′
TNF-α	Forward	5′-CCAGACCCTCACACTCAGATC-3′
	Reverse	5′-CACTTGGTGGTTTGCTACGAC-3′
MMP-9	Forward	5′-CATTCGCGTGGATAAGGAGT-3′
	Reverse	5′-CACTGCAGGAGGTCGTAGG-3′
MMP-12	Forward	5′-TTGTGGATAAACACTACTGGAGGT-3′
	Reverse	5′-AAATCAGCTTGGGGTAAGCA-3′
VEGF	Forward	5′-AGACGGACACACATGGAGGT-3′
	Reverse	5′-AAAGACTCAATGCATGCCAC-3′
GLUT1	Forward	5′-CCAGCTGGGAATCGTCGTT-3′
	Reverse	5′-CAAGTCTGCATTGCCCATGAT-3′
HK2	Forward	5′-TGATCGCCTGCTTATTCACGG-3′
	Reverse	5′-AACCGCCTAGAAATCTCCAGA-3′
G6pdx	Forward	5′-GTCCAGAATCTCATGGTGCTGA-3′
	Reverse	5′-GCAATGTTGTCTCGATTCCAGA-3′
PKM2	Forward	5′-TCGCATGCAGCACCTGATT-3′
	Reverse	5′-CCTCGAATAGCTGCAAGTGGTA-3′
Atp5b	Forward	5′-TGAGAGAGGTCCTATCAAAACCA-3′
	Reverse	5′-CACCAGAATCTCCTGCTCAAC-3′
SREBP1	Forward	5′-GCAGACCCTGGTGAGTGG-3′
	Reverse	5′-GTCGGTGGATGGGCAGTTT-3′
FASN	Forward	5′-TTGCTGGCACTACAGAATGC-3′
	Reverse	5′-AACAGCCTCAGAGCGACAAT-3′
ACCα	Forward	5′-CTGACGTATACTGAACTGGTGTTGGATG-3′
	Reverse	5′-TTTCCAGGCTACCATGCCAATCTC-3′
MGLL	Forward	5′-TCGGAACAAGTCGGAGGT-3′
	Reverse	5′-TCAGCAGCTGTATGCCAAAG-3′
CPT1α	Forward	5′-GACTCCGCTCGCTCATTC-3′
	Reverse	5′-TCTGCCATCTTGAGTGGTGA-3′
MCAD	Forward	5′-AGTACCCTGTGGAGAAGCTGAT-3′
	Reverse	5′-TCAATGTGCTCACGAGCTATG-3′
ACOX1	Forward	5′-GCCAAGGCGACCTGAGTGAGC-3′
	Reverse	5′-ACCGCAAGCCATCCGACATTC-3′
Cyclophilin A	Forward	5′-GAGCTGTTTGCAGACAAAGTTC-3′
	Reverse	5′-CCCTGGCACATGAATCCTGG-3′
